# Prevalence and characteristics of sleep apnoea in patients with stable heart failure: Results from a heart failure clinic

**DOI:** 10.1186/1471-2466-10-9

**Published:** 2010-03-03

**Authors:** Susana Ferreira, Anabela Marinho, Marta Patacho, Elisabete Santa-Clara, Cristina Carrondo, João Winck, Paulo Bettencourt

**Affiliations:** 1 Internal Medicine Department, São João Hospital, Oporto Medical University, Cardiovascular Research Unit, Alameda Professor Doutor Hernâni Monteiro, 4200-319 Porto, Portugal; 2 Pneumology Department, São João Hospital, Oporto Medical University, Cardiovascular Research Unit, Alameda Professor Doutor Hernâni Monteiro, 4200-319 Porto, Portugal

## Abstract

**Background:**

Heart failure (HF) and sleep apnoea (SA) association has been recognized but whether it results from confounding factors (hypertension, ischaemia, obesity) remains unclear.

We aimed to determine the prevalence of SA in HF and to identify potential risk factors for SA in HF population.

**Methods:**

We prospectively evaluated 103 patients with stable HF on optimized therapy. In-laboratory polysomnography was performed. Type and severity of SA were defined according international criteria. Demographic, anthropometric and clinical characteristics were collected. Continuous data are expressed as median and interquartile range.

**Results:**

SA was found in 72.8%, moderate to severe in a significant proportion (apnoea-hypopnoea index ≥ 15- 44.7% of all patients) and predominantly obstructive (60.0% of patients with SA). Most patients were non-sleepy (Epworth < 10- 66%). SA patients were predominantly men (85.3 vs 60.7%, p-0.015), had larger neck (38.0 (35.0-42.0) vs 35.0 (33.2-38.0) cm, p-0.003), severe systolic dysfunction, (63.9 vs 33.3%, p-0.018), left ventricle (LV) hypertrophy (16.2 vs 0.0%, p-0.03), LV and left atria (LA) dilatation (49.0 (44.0-52.0) vs 42.0 (38.0-48.0) mm, p < 0.001; 60.0 (54.0-65.0) vs 56.0 (52.0-59.0) mm, p-0.01). However, only LA diameter was an independent predictor of SA. Higher body-mass index (BMI) was associated with moderate to severe SA. Patients with obstructive SA had larger neck and a trend for higher BMI, snoring and sleepiness. Hypocapnia was not associated with central SA.

**Conclusions:**

In our HF population, SA was prevalent, frequently asymptomatic and without characteristic risk factors. Unlike previously reported, obstructive SA was the predominant type. These results suggest that SA is underdiagnosed in HF and there is a possible correlation between them, independent of confounding factors. Recent advances in HF therapy might influence prevalence and type of SA in this population.

## Background

Heart failure (HF) and sleep apnoea (SA) are prevalent, have significant morbimortality and a huge economic burden, making them public health problems [1-5].

SA has been associated with cardiovascular disease [6-8] and an independent correlation has been established with hypertension [9]. However, there is less evidence for HF [10-18]. A high prevalence of SA has been reported in HF populations (40-70%) but there is a limited number of studies and different diagnostic criteria and methods were used [7,13-18]. They evaluated heterogeneous populations, with small number of patients, mainly men. Also, there are several confounding factors (hypertension, ischaemic heart disease, obesity), making difficult to establish an independent relationship [10-12].

Most studies have described an association between HF and central type of SA [15,16,19-21]. It has been suggested that central SA may be a consequence of HF rather than a cause and it could be improved with optimization of HF therapy [19]. Obstructive SA has been considered a cardiovascular risk factor [6,7]. Nevertheless, both types of SA could increase the likelihood of having HF (OR 2.38, 95%CI 1.22-4.62) [7] and may be associated with worse outcome [20-22].

HF continues to be devastating despite advances in pharmacotherapy [3,4]. It is crucial to identify and treat comorbidities which may contribute to its progression. HF studies have focused in the awake patient, probably because sleep has been seen as a cardiovascular rest state [10-12]. However, SA interrupts it and may contribute for progression of HF by several mechanisms: activation of neurohumoral systems (sympathetic, renin-angiotensin, natriuretic peptides); recurrent hypoxemia leading to myocardial ischaemia; successive negative deflections in intrathoracic pressure leading to afterload increase, preload decrease and lower stroke volume; inflammatory activation, prothrombotic state and endothelial dysfunction [10-12]. Standard treatment for SA (CPAP) has been shown to improve symptoms and cardiac function in HF, but there is conflicting results on its impact on prognosis [23,24].

The aims of this study were to determine the prevalence of SA in a stable HF population, diagnosed and treated according updated international recommendations [1,2]. To evaluate the characteristics of HF patients with SA which may help to identify the population at risk in clinical practice.

## Methods

### Patients and study protocol

Between March 2005 and July 2007, 103 patients were consecutively recruited from a HF clinic, in an university hospital.

They had to be stable and on optimized therapy for inclusion in the study. HF was defined according to the European Society of Cardiology [1]. Patients were considered stable if there were no changes in New York Heart Association (NYHA) class or medications within the last month. Exclusion criteria were: unstable HF; acute coronary syndrome in the previous 3 months; previous diagnosis of SA or hypoventilation syndrome; treatment with positive pressure ventilation (CPAP or BiPAP) in the previous 3 months. Only 5 patients were excluded because they were already on non-invasive ventilation.

Each patient was evaluated and completed Epworth Sleepiness Scale (ESS) which is validated in Portuguese language [25]. Anthropometric features were recorded by the same observer during the study (body-mass index (BMI), waist/hip, neck circumference, thyromental distance and Mallampati classification). Mallampati scoring is a simple non-invasive method to assess the airway. It is commonly used by the anesthesiologists to predict the difficulty of endotracheal intubation, but it has been correlated with the presence and severity of obstructive SA [26].

Polysomnography was performed, using a multichannel polygraph (SomnoStar CephaloPro, SensorMedics^®^), monitored and manually scored by two technicians who were the same during all the study. We recorded electro-encephalogram, electro-oculogram, submental electromyogram, heart rate and body position. Thoraco-abdominal excursions were registered using respiratory inductive plethysmography. Nasal airflow was measured using a thermocouple and nasal pressure transducer. Oxyhemoglobin saturation was recorded using a pulse oximeter.

The following tests were also obtained: complete blood count and biochemistry, plasma NT-proBNP levels (immunoassay DadeBehring^®^), arterial blood gas and spirometry (in the morning following polysomnography). Echocardiography was standardized across all patients and time separation between polysomnography and echocardiography was 6 months to 1 year.

This protocol was approved by the local ethics committee and patients signed an informed consent form.

### Diagnosis and classification of sleep apnoea

Sleep apnoea was defined according to the American Academy of Sleep Medicine [27]. An apnoea was defined as complete cessation of inspiratory flow for 10 seconds or more. Hypopnoea was defined as a reduction of airflow or thoraco-abdominal excursions lasting 10 seconds or more and associated with at least 4% drop in oxygen saturation and/or arousal. An arousal was defined as appearance of alpha-waves on electro-encephalogram for at least 3 seconds. Obstructive SA was defined as the absence/reduction of airflow in the presence of thoraco-abdominal excursions. Central SA was defined by absence of respiratory effort. Cheyne-Stokes respiration was considered in the presence of at least three consecutive cycles of a cyclical crescendo-decrescendo change in breathing amplitude.

Severity of SA was defined according to the number of apnoeas and hypopnoeas per hour of sleep - apnea-hypopnea index (AHI): 5-14 - mild; 15-29 - moderate; ≥ 30 - severe.

Type of SA was defined according to the most prevalent events: ≥ 50% obstructive - obstructive SA; ≥ 50% central - central SA; no prevalent type - mixed. Sleep studies were also scored to assess the presence of Cheyne-Stokes respiration.

### Data analysis

We determined the total prevalence of SA in our HF population and according to severity and type of SA. Given the small number of patients with pure central SA, for the purpose of this study, they were associated to patients with mixed type.

We compared the characteristics of patients without SA (AHI < 5) and: with SA (AHI ≥ 5); with moderate to severe SA (AHI ≥ 15); with obstructive SA; with central/mixed SA.

Continuous variables were reported as median and interquartile range and Mann-Whitney test was used for comparison between two groups. For categorical variables, χ^2 ^or Fisher test were used. Multiple logistic regression was used to identify the variables that were independently associated with the presence of SA. p value < 0.05 was considered significant.

## Results

### Prevalence and type of sleep apnoea

**Figure 1:**
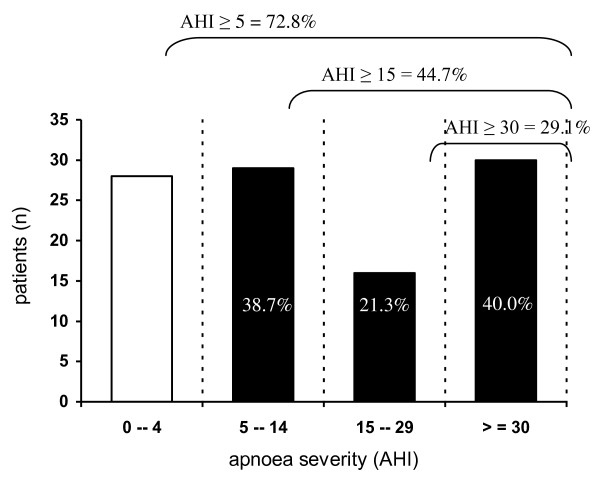
**Prevalence and severity of sleep apnoea in heart failure**. AHI- apnoea-hypopnoea index.

According to the defined criteria, there were 75 patients (72.8%) with SA (AHI ≥ 5) and 44.7% had moderate to severe SA (AHI ≥ 15) (Figure 1). The median AHI was 17.6/hour (9.3-42.5). Among patients with SA, most had moderate to severe SA (61.3%, n = 46), with median AHI of 40.4 (26.8-61.2). A significant proportion had severe SA (n = 30, 40% of patients with SA and 29.1% of all patients) (Figure 1).

**Figure 2:**
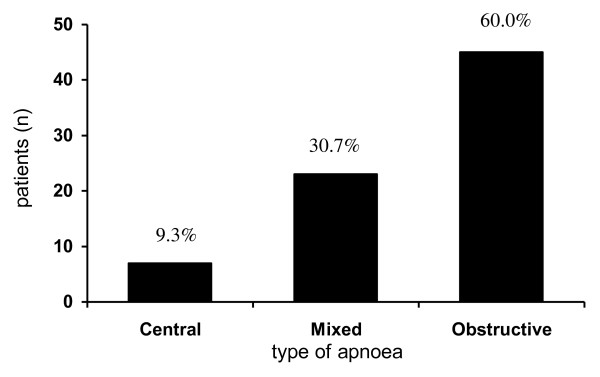
**Type of sleep apnoea in heart failure**.

Obstructive SA was the most prevalent type (60.0%, n = 45) and only 9.3% of patients (n = 7) had pure central SA (Figure 2). Both types of SA (mixed SA) were present in 23 patients (30.7%) (Figure 2). Cheyne-Stokes respiration was noted in 19 patients (25.6%), independently of the predominant type of SA.

### Patients characteristics and comparison between groups

Patients characteristics are shown in Tables 1, 2 and 3 (in the additional files section).

**Table 1:** Risk factors for sleep apnoea in heart failure.

	Total (N = 103)	With SA (N = 75)	Without SA (N = 28)	p value
**Age (years)**	66.0 (57.0-75.0)	66.0 (57.0-75.0)	67.5 (60.2-74.8)	0.70
**Male (%)**	78.6	85.3	60.7	0.015
**BMI (Kg/m**^2^**)**	27.2 (23.6-30.8)	27.0 (24.0-31.0)	27.4 (22.5-29.6)	0.25
**Waist/hip**	0.97 (0.92-1.00)	0.98 (0.92-1.02)	0.96 (0.93-1.0)	0.32
**Neck circumference (cm)**	37.0 (35.0-41.0)	38.0 (35.0-42.0)	35.0 (33.2-38.0)	0.003
**Thyromental distance (cm)**	7.0 (6.0-8.0)	7.0 (6.0-8.0)	7.0 (5.2-8.0)	0.56
**Mallampati C-D (%)**	58.3	60.3	53.6	0.70
**Smoking (%)**	51.5	53.3	46.4	0.69
**Drinking (%)**	41.7	46.7	28.6	0.15
**Snoring (%)**	71.8	77.3	57.1	0.08
**ESS (score)**	5.5 (2.8-10.0)	7.0 (3.0-10.0)	4.0 (1.0-9.0)	0.08

AHI- apnoea-hypopnoea index; BMI- body-mass index; ESS- Epworth sleepiness scale.

Continuous data are expressed as median and interquartile range.

**Table 2:** Cardiovascular characteristics in heart failure patients with and without sleep apnoea.

	Total (N = 103)	With SA (N = 75)	Without SA (N = 28)	p value
**Hypertension (%)**	55.3	56.0	53.6	1.00
**Ischaemic aetiology (%)**	54.4	54.7	53.6	1.00
**Atrial fibrillation (%)**	34.3	36.5	28.6	0.60
**NYHA II-III vs I (%)**	61.8	67.6	46.4	0.08
**Echocardiography**				
**Severe LVSD (%)**	56.3	63.9	33.3	0.018
**LVH (%)**	12.1	16.2	0.0	0.03
**LVEDD (mm)**	59.0 (54.0-64.3)	60.0 (54.0-65.0)	56.0 (52.0-59.0)	0.01
**LA (mm)**	47.0 (41.0-51.0)	49.0 (44.0-52.0)	42.0 (38.0-48.0)	< 0.001
**PSAP (mmHg)**	48.0 (40.0-52.8)	50 (40-53)	45 (38-52)	0.46
**NT-proBNP (pg/mL)**	1260.6 (552.3-2493.2)	1317.6 (705.9-2218.9)	1137.7 (232.9-2718.2)	0.51

AHI- apnoea-hypopnoea index; NYHA- New York Heart Association; LVSD- left ventricle systolic dysfunction; LVH- left ventricle hypertrophy; LVEDD- left ventricle end-diastolic diameter; LA- left atria; PSAP- pulmonary systolic arterial pressure; RV- right ventricle.

Continuous data are expressed as median and interquartile range.

**Table 3:** Sleep characteristics in heart failure patients with and without sleep apnoea

	Total (N = 103)	With SA (N = 75)	Without SA (N = 28)	p value
**AHI (n/h)**	10.4 (4.6-30.5)	17.6 (9.3-42.5)	1.9 (0.6-4.1)	< 0.001
**Total sleep time (TST, min)**	246.0 (189.5-333.0)	246.2 (189.5-334.0)	222.0 (131.5-301.5)	0.34
**Sleep (% TST)**				
**Stage 1**	15.5 (10.0-23.4)	17.5 (12.7-27.4)	11.6 (5.4-15.3)	< 0.001
**Stage 2**	60.4 (49.8-71.2)	60.4 (50.3-72.0)	60.9 (46.6-70.4)	0.58
**Stages 3 and 4**	10.9 (1.0-20.3)	6.9 (0.3-15.1)	19.3 (13.4-31.5)	< 0.001
**REM**	8.0 (3.1-12.7)	8.4 (3.8-13.1)	6.4 (0.0-10.9)	0.17
**NREM**	92.1 (87.4-97.2)	91.6 (87.1-96.4)	93.6 (89.100.0)	0.22
**Arousals (n/h)**	6.1 (3.1-10.4)	7.2 (4.0-11.0)	3.8 (1.0-7.3)	0.02
**Mean SO2 (%)**	93.0 (91.0-95.0)	93.0 (90.0-94.0)	94.0 (92.0-96.0)	0.02
**Lowest SO2 (%)**	81.0 (76.0-85.0)	81.0 (71.2-85.0)	85.0 (79.0-88.0)	0.006
**SO2 < 90% (% TST)**	7.7 (2.0-27.6)	15.6 (3.7-39.8)	2.0 (0.2-6.2)	< 0.001

AHI- apnoea-hypopnoea index; SO2- oxyhemoglobin saturation.

Continuous data are expressed as median and interquartile range.

### According to the severity of sleep apnoea

Patients with SA (AHI ≥ 5) were predominantly men. They had larger neck, but no other significant differences in craniofacial characteristics. Total population was overweight but BMI was not associated with SA. Most patients were non-sleepy (ESS < 10-66%) and snoring but there was a trend toward higher ESS score and snoring in patients with SA (Table 1).

More than half of patients had severe left ventricular systolic dysfunction but it was more prevalent in patients with SA. Hypertension was equally prevalent, but left ventricle hypertrophy (LVH), left ventricle (LV) and atria (LA) dilatation were more common in patients with SA. There were no significant differences in pulmonary arterial pressure and right heart dilatation despite higher values were noted in the SA group (Table 2). Most patients were optimally treated according international recommendations and there were no significant differences between patients with and without SA: furosemide - 95.1%, 76.3 ± 44.2 mg; ACE-inhibitors - 95.1%, 13.0 ± 9.0 mg (lisinopril equivalent); beta-blockers - 90.3%, 30.9 ± 19.9 mg (carvedilol equivalent); spironolactone - 42.7%, 7.9 ± 12.9 mg.

Sleep efficiency was low but it was not significantly different between patients with and without SA (52.0 vs 57.0%, p-0.67). More than half of all patients had obstructive syndrome (FEV1/FVC < 70%- 55.1%) but prevalence was not significantly different in patients with and without SA (59.2 vs 44.4%, p-0.28). Patients with SA had more nocturnal desaturation and lower oxygen saturation (Table 3), despite having similar and normal awake blood gases (PaO2- 81.8 (73.6-90.6) vs 81.6 (76.0-87.2), p-0.87; PaCO2- 41.0 (37.7-43.6) vs 41.7 (39.2-44.2) mmHg, p-0.47).

When compared to patients without SA, those with moderate to severe SA (AHI ≥ 15) were similar to patients with AHI ≥ 5: predominantly men- 91.3 vs 60.7%, p-0.004; larger neck - 38.0 (35.0-44.0) vs 35.0 (33.2-38.0) cm, p-0.003; severe LV systolic dysfunction- 68.2 vs 33.3%, p-0.01; LV and LA dilatation: LV end-diastolic diameter- 61.0 (57.0-65.0) vs 56.0 (52.0-59.0) mm, p-0.008; LA diameter- 49.0 (44.0-52.0) vs 42.0 (38.0-48.0) mm, p < 0.001; more light sleep: stage 1- 20.0 (13.5-32.7) vs 11.6 (5.4-15.3) %, p < 0.001, sleep stages 3 and 4- 2.7 (0.0-10.4) vs 19.3 (13.4-31.5) %, p < 0.001. However, higher BMI was also associated with moderate to severe SA (29.1 (25.7-33.5) vs 27.4 (22.5-29.6) Kg/m^2^, p-0.05).

In multivariate analysis, when adjusted for gender, BMI, neck circumference, NYHA class II-III, severe systolic dysfunction, LV diastolic diameter and LA diameter per body surface, only LA diameter (each milimeter increase) was an independent predictor of SA in HF patients (OR 1.11, 95%CI 0.99-1.26). Male gender, BMI and NYHA class II-III were independent predictors of moderate to severe SA (respectively: OR 6.70, 95%CI 1.64-27.28; OR 1.12, 95%CI 1.02-1.24; OR 2.97, 95%CI 1.08-8.17).

### According to the type of sleep apnoea

When compared to patients without SA, those with obstructive type had larger neck (39.0 (35.0-43.5) vs 35.0 (33.2-38.0) cm, p-0.002). There was a trend for higher BMI (29.1 (24.5-33.3) vs 27.4 (22.5-29.6), p-0.09), snoring (80.0 vs 57.1%, p-0.07) and higher ESS score (ESS- 7 (4.0-12.0) vs 4 (1.0-9.0), p-0.07).

Patients with central/mixed SA were predominantly men (96.7 vs 60.7, p-0.002) and had a trend for larger neck (36.0 (35.0-40.0) vs 35.0 (33.2-38.0) cm, p-0.05).

Severe LV systolic dysfunction was significantly associated with obstructive SA and there was a trend for an association with central/mixed SA (respectively: 66.7%, p-0.02; 60.0%, p-0.09). LA diameter was related to both types of SA (obstructive- 48 (43.5-53.0) mm, p-0.002; central/mixed- 49.0 (44.5-51.5) mm, p-0.001).

When we compared different patterns of SA (obstructive vs central/mixed SA), patients with obstructive SA had a trend for higher BMI (29.1 (24.5-33.3) vs 26.1 (23.4-29.2) Kg/m^2^, p-0.08). Despite they had larger neck than patients without SA, their neck circumference was not significantly different from patients with central/mixed SA (39.0 (35.0-43.5) vs 36.0 (35.0-40.0) cm, p-0.16). Other craniofacial characteristics were also similar (Mallampati C and D- 60.0 vs 60.7%, p-1.0; thyromental distance- 7.0 (6.0-8.0) vs 7.0 (6.0-8.0) cm, p-0.41). Snoring was equally prevalent (80.0 vs 73.3%, p-0.19).

Patients with central/mixed pattern had more severe SA (AHI-32.9 (10.8-71.9) vs 15.7 (8.9-29.6), p-0.02) and shorter total sleep time (215.5 (175.0-247.0) vs 278.5 (228.5-354.5) minutes, p-0.001). They were significantly older (72.0 (62.8-78.2) vs 63 (53.5-70.5) years, p-0.008) and mostly men (96.7 vs 77.8%, p < 0.001). Ischaemic aethiology was more prevalent in this group (70.0 vs 44.4%, p-0.015).

Severe LV systolic dysfunction was not associated to a specific pattern of SA (obstructive- 66.7 vs central/mixed- 60.0%, p-0.28). Spironolactone was more often prescribed in patients with obstructive SA (51.1 vs 33.3%, p-0.02). Other therapies were similar in the two groups.

## Discussion

In this prospective study of well-defined, severe, stable HF outpatients, on optimized therapy, we found a high prevalence of SA (AHI ≥ 5- 72.8%, AHI ≥ 15- 44.7%), independent of clinical suspicion or symptoms (ESS < 10- 66%). This emphasizes the relevance of the association between those public health problems and question the need for screening of SA in HF population.

It is in agreement with previous studies, but there is a high variability in the reported prevalence of SA in HF (40-70%) [13-18]. Possible explanations are the small number of patients involved in most studies, predominantly men, the heterogeneity of populations and the different used diagnostic criteria and methods. Our study is one of the largest in this area, using more recent sleep sensors technology and international diagnostic criteria [27]. It reports data from a heart failure clinic, including patients with significant comorbidities. Most patients were referred to the clinic after an acute HF episode. These patients, although not representative of the general population, have some characteristics similar to Heart Failure in the general population. Epidemiological studies performed in Portugal and Spain reported a medium age of 64 and 65 years in Heart Failure patients [28,29]. Our study included 55% of patients older than 65 years (mean age 66 years), higher than other studies previously published [13-18]. Mared et al reported a high prevalence of Cheyne-Stokes respiration in an older HF population (mean age 73 years) [30]. However, patients were hospitalised for decompensated HF and not stable and ambulatory as in our study.

Unlike previously reported, in our HF population, SA was predominantly obstructive.

Central SA has been seen as a consequence of HF, correlated with its severity and prognosis [19-21]. However, this could not explain our results since we had a high prevalence of severe LV systolic dysfunction in the total population (56.3%), significantly associated with SA (63.9 vs 33.3, p-0.018) but not to central/mixed type. Also, in contrast to other studies [15,16], SA was predominantly obstructive (60%). Only 9.3% of patients had pure central SA and a significant proportion had both types (30.7%).

Despite the severity of HF, patients were stable and on optimized therapy which could contributed to lower the prevalence of central SA in our study. The management of HF has evolved in last decade, with widespread use of ACE-inhibitors and beta-blockers [1-4]. The new pharmacotherapy has significantly improved prognosis of HF [3,4], but the impact in the prevalence and type of SA has not been evaluated [16,17,31,32]. Two recent studies have shown that HF patients taking beta-blocker have a lower prevalence and severity of SA [31,32]. In contrast to some of the previous studies, our patients were receiving optimal treatment for HF (beta-blocker- 90.3%, ACE-inhibitors- 95.1%, spironolactone- 42.7%). In some of the studies that reported predominance of central SA, use of beta-blocker was lower (10 and 78%) [15,16].

Javaheri et al studied 100 patients with severe stable HF [15]. Prevalence of moderate to severe SA (AHI ≥ 15) was similar to our population (49.0 vs 44.7%) but central SA was the predominant type (37.0 vs 12.0%). However, only men were included and the investigation started in nineties, when current HF therapy was not widespread (beta-blocker-10%, ACE-inhibitors- 91%). More recently, Vazir et al reported similar results [16] in a HF population with improved treatment (beta-blocker- 78%, ACE-inhibitors- 98%, spironolactone- 49%).

Nevertheless, other studies found a high proportion of obstructive SA, in according to our results [14,17,18]. In a population of 53 outpatients with stable HF, prevalence of SA (AHI > 10) was 68%, predominantly obstructive (53%) [14]. Oldenburg et al studied 700 patients and found 76% prevalence of SA (AHI ≥ 5), 36% obstructive [17]. Schulz and colleagues studied 203 patients and reported a prevalence of 71% (AHI > 10), 43% obstructive [18]. In the two last studies, diagnosis was based in polygraphy which can underestimate AHI. As a consequence, the prevalence and severity of SA might even be higher.

Our patients were overweight which could contribute to the predominance of obstructive SA. However, BMI was not different in patients with and without SA and median BMI was similar to studies where central SA was predominant [15,16]. Neck circumference was significantly greater in patients with SA which could contribute to the predominance of the obstructive type. Ferrier et al studied a HF population with a larger neck (41.0 vs 37.0 cm) and found similar results [14]. Craniofacial anthropometric characteristics were not mentioned in other studies [13,15-18].

Characteristics that could help to identify HF patients with SA have been described: male gender for both types of SA; age > 60 years, atrial fibrillation and hypocapnia for central SA; for obstructive SA, higher BMI in men and age > 60 years in women [13]. However, this study was retrospective, patients were recruited from a population referred for suspected SA and asymptomatic patients were excluded. In the study by Javaheri et al, NYHA class III, low LV ejection fraction, atrial fibrillation and hypocapnia were associated with central SA [15]. Obesity and snoring were risk factors for obstructive SA. Similar results were reported by other authors [14,16,17].

We did not find characteristic risk factors for SA in patients with HF. Male gender, larger neck, severe systolic dysfunction, LV hypertrophy, LV and LA dilatation were significantly associated with SA. However, in multivariate analysis, only LA diameter was an independent predictor of SA. Its significance is uncertain, but LA dilatation could be an early marker of hypertension and diastolic dysfunction which have been independently associated with SA [9].

Patients with obstructive SA had larger neck and a trend for higher BMI, snoring and higher ESS score which is in agreement with literature [13]. Patients with central/mixed SA were predominantly men and older. Unlike previously reported [13,15], awake hypocapnia was not associated with central SA.

As previously described [14], in our population NT-proBNP levels were not different in all groups but patients with SA had more severe LV systolic dysfunction. Severe systolic dysfunction was not associated to a specific pattern of SA. Other authors found higher levels of NT-proBNP in patients with SA but no difference in LV systolic dysfunction [16].

In our study, sleep efficiency was unsatisfactory. This could be explained by the "first-night effect", resulting from insomnia related to a different environment. However, as sleep efficiency was not different in patients with or without SA, it didn't interfere with the results.

Patients with SA had more sleep fragmentation and nocturnal desaturation but not increased daytime sleepiness, in accordance with other studies [15-18]. This suggests that subjective daytime sleepiness measured by ESS is not useful in screening of SA in HF patients, but deleterious effects of inefficient sleep are present.

We are aware of some limitations to our study. The number of patients in each group is relatively small but, to our knowledge, it is one of the largest studies in the literature. Other studies involved a much larger number of patients but they were retrospective and in a HF population with suspected SA [13]. Unlike in our study, diagnosis was based in polygraphy [17,18] which is not still validated in HF population [33]. The small number of patients with pure central SA and its association with mixed type, could limit interpretation of data.

## Conclusions

Prevalence of sleep apnoea was high in patients with stable HF despite optimized therapy (72.8%). Most were non-sleepy (ESS < 10- 66%). Unlike has been usually reported, obstructive SA was the predominant type (60%). Male gender, larger neck, severe systolic dysfunction, LV hyperthrophy, LV and LA dilatation were associated with SA but only LA diameter was an independent predictor.

These results suggest that SA is underdiagnosed in HF because it is frequently asymptomatic and characteristics risk factors are not present in most patients. There is a possible correlation between HF and SA independent of most known confounding factors. Recent advances in HF therapy which improve cardiac function and outcome might influence prevalence and type of SA in this population. If standard treatment for SA is shown to improve prognosis in patients with HF, screening would be recommended.

## Competing interests

The authors declare that they have no competing interests.

## Authors' contributions

SF conceived of the study, participated in its design and coordination, collected demographic, anthropometric and clinical information, performed the statistical analysis, participated in the sequence alignment and drafted the manuscript. AM participated in its design, collected demographic and clinical information, review polysomnography, participated in the sequence alignment and drafted the manuscript. MP participated in its design, collected demographic and clinical information, participated in the sequence alignment and drafted the manuscript. ESC carried out the polysomnography, spirometry and arterial blood gas. CC carried out the polysomnography, spirometry and arterial blood gas. JW participated in the design of the study and coordination, in the sequence alignment and drafted the manuscript. PB participated in the design of the study and coordination, in the sequence alignment and drafted the manuscript. All authors read and approved the final manuscript.

## Pre-publication history

The pre-publication history for this paper can be accessed here:

http://www.biomedcentral.com/1471-2466/10/9/prepub
